# First Demonstration of Functional Task Performance Using a Sonomyographic Prosthesis: A Case Study

**DOI:** 10.3389/fbioe.2022.876836

**Published:** 2022-05-04

**Authors:** Susannah M. Engdahl, Samuel A. Acuña, Erica L. King, Ahmed Bashatah, Siddhartha Sikdar

**Affiliations:** ^1^ Department of Bioengineering, George Mason University, Fairfax, VA, United States; ^2^ Center for Adaptive Systems of Brain-Body Interactions, George Mason University, Fairfax, VA, United States

**Keywords:** upper limb, prosthesis control, gesture recognition, ultrasound imaging, sonomyography, rehabilitation, functional tasks

## Abstract

Ultrasound-based sensing of muscle deformation, known as sonomyography, has shown promise for accurately classifying the intended hand grasps of individuals with upper limb loss in offline settings. Building upon this previous work, we present the first demonstration of real-time prosthetic hand control using sonomyography to perform functional tasks. An individual with congenital bilateral limb absence was fitted with sockets containing a low-profile ultrasound transducer placed over forearm muscle tissue in the residual limbs. A classifier was trained using linear discriminant analysis to recognize ultrasound images of muscle contractions for three discrete hand configurations (rest, tripod grasp, index finger point) under a variety of arm positions designed to cover the reachable workspace. A prosthetic hand mounted to the socket was then controlled using this classifier. Using this real-time sonomyographic control, the participant was able to complete three functional tasks that required selecting different hand grasps in order to grasp and move one-inch wooden blocks over a broad range of arm positions. Additionally, these tests were successfully repeated without retraining the classifier across 3 hours of prosthesis use and following simulated donning and doffing of the socket. This study supports the feasibility of using sonomyography to control upper limb prostheses in real-world applications.

## 1 Introduction

Upper limb prostheses are abandoned by users at an astonishing rate despite the significant functional deficits imposed by the loss of an upper limb (Biddiss E. A. and Chau T. T., 2007). As much as 98% of users who have rejected a prosthesis believe they are equally or more functional without one, although 74% of those who have abandoned a prosthesis would reconsider this decision if improvements were made (Biddiss E. and Chau T., 2007). Consequently, advancements in upper limb prostheses have focused on addressing predominant user concerns relating to comfort and functionality (Biddiss E. and Chau T., 2007; Smail et al., 2020). In particular, significant effort has been dedicated towards enabling intuitive control of multi-articulated prosthetic hands (e.g. (Kuiken et al., 2009; Weir et al., 2009; Hargrove et al., 2018; Resnik et al., 2018a; Simon et al., 2019; Vu et al., 2020b),), which might facilitate improved functional outcomes (Kuiken et al., 2016; Resnik et al., 2018b).

Prosthetic hands are typically controlled via the electrical activity of muscle contractions in the residual limb. Myoelectric systems can record and decode these electromyographic (EMG) signals to predict a user’s intended configuration of their prosthetic hand. Grasp prediction relies on classification algorithms to compare features of incoming EMG signals to sets of previously-collected EMG signals for known hand configurations (i.e., supervised learning using training data). Time-domain or frequency-domain features of the EMG signals can be used for training and classification, with varying classification accuracy (Fang et al., 2020). Unfortunately, using EMG sensors on a large set of individual muscles within the residual limb is challenging because crosstalk between sensors restricts the number of independent EMG signals that are actually available (Vu et al., 2020a). This problem restricts the degrees of freedom within the hand that may be controlled via EMG (Graimann and Dietl, 2013). However, a user might require a rich set of control signals to enable more intuitive control of their prosthetic hand (e.g., for independent actuation of each degree of freedom).

Sonomyography (SMG) is an alternative approach for prosthesis control that relies on ultrasound imaging to sense muscle deformation within the residual limb during voluntary movement (Sikdar et al., 2014). Similar to EMG control, SMG control employs a supervised learning framework, using classification algorithms to compare features of ultrasound signals to training data. However, because ultrasound enables spatiotemporal characterization of both superficial and deep muscle activity, crosstalk can be avoided. As a result, it is possible to derive a rich set of prosthesis control signals that may better account for the independent contributions of individual muscles. Ultrasound images of forearm muscle tissue from a single transducer have enough unique spatiotemporal information for classification algorithms to differentiate between various hand grasps. For example, we previously used SMG to identify five individual digit movements in able-bodied individuals with 97% cross-validation accuracy (Sikdar et al., 2014) and fifteen complex hand grasps with 91% cross-validation accuracy (Akhlaghi et al., 2016). We also found that, with minimal training required, SMG can identify five grasps for individuals with upper limb loss with 96% cross-validation accuracy (Dhawan et al., 2019; Engdahl et al., 2022). Thus, it is not surprising that SMG is becoming a promising option for hand gesture recognition and prosthesis control for able-bodied individuals (Chen et al., 2010; Shi et al., 2010; Yang et al., 2019, 2020) and individuals with upper limb loss (Zheng et al., 2006; Hettiarachchi et al., 2015; Baker et al., 2016; Dhawan et al., 2019). However, it is still unclear if SMG is a practical way to control an upper limb prosthesis for real-time functional task performance.

There are several reasonable concerns regarding the feasibility of using SMG to control an upper limb prosthesis in real-world settings, where classification accuracy can degrade due to a variety of physiological, physical, and user-specific factors (Kyranou et al., 2018). For example, ultrasound imaging may inherently be too sensitive to changes in arm position and socket loading during task performance. Even minor changes to the imaging angle can drastically affect an acquired ultrasound image and cause the classifier to misidentify the user’s intended hand gesture. Unintended hand movements due to misclassification may lead to reduced task completion rates, slower task performance, increased temporal variability, and increased cognitive load (Chadwell et al., 2021). Thus, grasp classification must be sufficiently stable under varying arm positions and loading conditions for the user to consistently achieve their desired hand grasps throughout the reachable workspace. Although classification training and testing are typically performed “offline” to avoid these confounding real-world factors and optimize signal quality, offline classification accuracy is not considered an adequate measure of real-time function (Li et al., 2010; Ortiz-Catalan et al., 2015). Real-time functional testing with a physical prosthesis is therefore crucial for demonstrating the viability of SMG as control modality. Some prior studies involving SMG have successfully implemented real-time virtual target-tracking tasks (Chen et al., 2010; Dhawan et al., 2019), as well as real-time control of virtual hands (Castellini et al., 2014; Baker et al., 2016) or benchtop robotic grippers (Bimbraw et al., 2020). However, these studies are not sufficient to demonstrate the viability of real-world prosthetic control using SMG.

The objective of this study was to investigate whether it is feasible for an individual with upper limb loss to perform functional tasks using a prosthesis controlled by SMG. Acknowledging the potential challenges introduced by operating a prosthesis in real-time, we examined real-time performance during tasks that required the user to select different grasps over a broad range of arm positions. We also examined the repeatability of task performance over 3 hours of continuous use and with simulated doffing and donning of the socket. As part of these investigations, we considered different classifier training strategies to account for changes to arm position and socket loading. Additionally, we quantified differences in the associated ultrasound images to contextualize classifier performance.

## 2 Methods

### 2.1 Participatory Study Design

We followed a participatory research design involving a single patient. Participatory research is intended to engage patients as equal partners with the research team following three primary principles: co-ownership and shared governance of the research, innovation by the participants, and giving primacy to the views of participants (Andersson, 2018). Thus, patients serve as co-researchers who actively play a role in the entire research process from study creation to completion, rather than simply serving as a test subject. Following these principles, our participant was involved with all stages of the research process. The participant’s perspective was extensively involved in establishing the study objectives, as well as designing and refining the methodology through iterative pilot testing. Upon completion of data collection, the participant also contributed to interpretation of the results and thus is included as an author.

### 2.2 Participant

The participant was a 30-year-old female with congenital bilateral limb absence. Both limbs were affected at the wrist disarticulation level. The participant reported use of single degree of freedom, direct control myoelectric prostheses with both limbs for 27 years, as well as right-hand dominance. The study was reviewed and approved by the Institutional Review Board at George Mason University. The participant provided written informed consent to participate in this study, and to include her data and identifiers in publications about the study.

### 2.3 Socket Design and Fit

**FIGURE 1:**
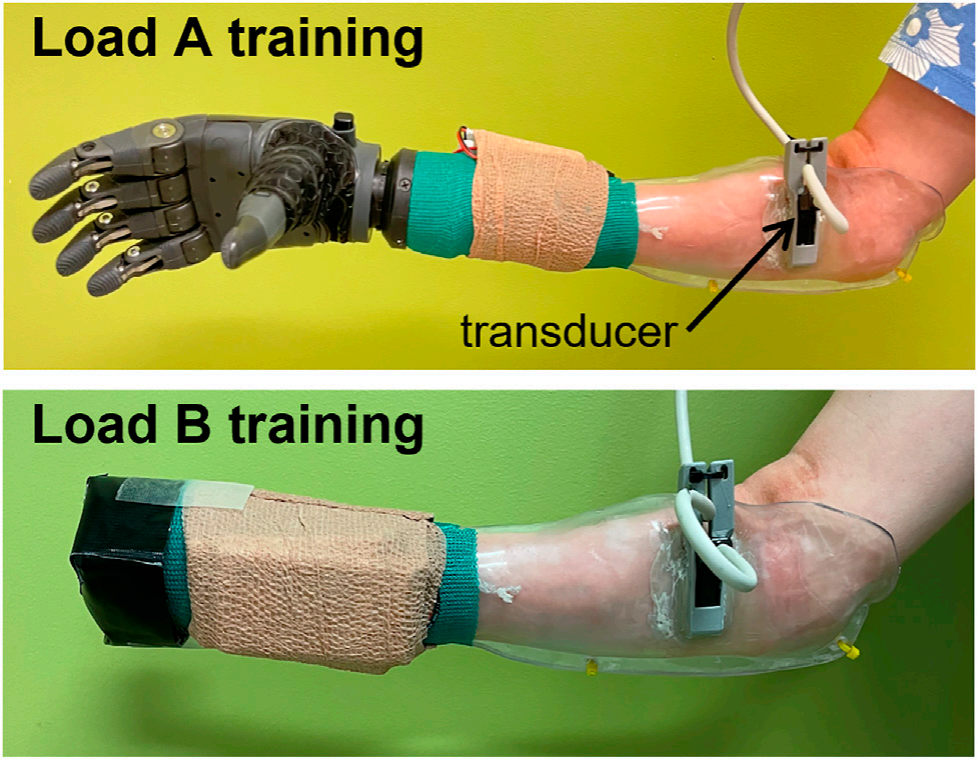
Classifier training was performed with all distal components attached during the *load A* condition and with a weight equal to the participant’s clinically-prescribed prosthetic hand during the *load B* condition.

The participant was fitted with thermoplastic test sockets on both residual limbs using supracondylar suspension. The sockets weighed 223 g. A TASKA Hand and Quick Disconnect Wrist (TASKA Prosthetics, Christchurch, New Zealand) and MC Standard Wrist Rotator (Motion Control Inc., Salt Lake City, UT) were mounted to the socket. The left hand was 19.3 cm long and weighed 567 g. The right hand was 20.4 cm long and weighed 680 g. The wrist rotator was 7 cm long and weighed 149 g. The batteries and on/off switch weighed 105 g. The resulting prosthesis was extremely long relative to the participant’s height (149.86 cm) and residual limb length (20.5 cm for the right limb and 19.3 cm for the left limb). It was also considerably heavier than the participant’s clinically-prescribed myoelectric prostheses, with much of the weight located distally due to the size of the TASKA Hand (Figure 1). The participant reported that the socket alone was well-fitted over the limb, but when the components were attached to the distal end of the socket (i.e., the TASKA Hand, wrist rotator, batteries, and on/off switch), the fit noticeably deteriorated.

### 2.4 Ultrasound Imaging

A low-profile, high-frequency, linear 16HL7 ultrasound transducer weighing 11 g was mounted on the socket via a custom 3D printed bracket (Figure 1). The transducer was positioned such that it made direct contact with the volar surface of the residual limb when the prosthesis was donned (i.e., over the forearm muscle tissue of the residual limb). We acquired ultrasound images using a clinical ultrasound system (Terason uSmart 3200T, Terason, Burlington, MA) and transferred them to a PC in real-time using a USB-based video grabber (DVI2USB 3.0, Epiphan Systems, Inc., Palo Alto, CA). The video grabber digitized the images at 8 bits/pixel. Using a custom MATLAB script (MathWorks, Natick, MA), we downscaled the ultrasound images to 100 × 140 pixels before processing with a classifier, as described below.

### 2.5 Classifier Training Conditions

We acquired a set of ultrasound images under various training conditions to be used as training data for a classifier (Table 1). For each acquisition, we instructed the participant to perform a forearm muscle contraction corresponding to a desired hand grasp and maintain this contraction for a specified duration. We also instructed the participant to perform contractions at a comfortable level and allowed her to rest between periods of classifier training to minimize fatigue. Note that the TASKA hand and wrist rotator were not active during classifier training.

**TABLE 1:** All training and testing conditions implemented in this study. Results from offline testing were used to select a subset of training conditions for functional testing.

Training Conditions		Testing Conditions			
*Arm Position*	*Socket Loading*	*Offline* ^a^	*Short-Term* ^b^	*Three-Hour* ^b^	*Donning/Doffing* ^b^
Static	Load A	Right arm	–	–	–
	Load B	Right arm	Right arm	–	–
Dynamic	Load A	Right arm	–	–	–
	Load B	Right arm	–	–	–
Continuous Dynamic	Load A	–	Right arm	–	–
	Load B	–	Both arms	Both arms	Both arms

a Outcome measures: Classification accuracy; SSIM.

b Outcome measures: Test scores; Number of transient bouts; Percent of frames classified as point.

We used linear discriminant analysis (LDA) classifiers to predict the user’s desired hand grasp from the acquired ultrasound training data. While more complex classification algorithms are possible, LDA classifiers are commonly used for myoelectric control given their simple implementation, strong classification performance, and computational efficiency (Fougner et al., 2011; Geng et al., 2012). As described below, we considered different classifier training strategies to account for changes to arm position and socket loading.

#### 2.5.1 Hand Grasps

We trained the classifiers to recognize a set of three intended hand grasps: *tripod* grasp, index finger *point*, and *rest*. Any repeatable muscle deformation pattern could be mapped to these intended hand grasps, therefore we asked the participant to choose a set of muscle contractions that would be easiest to perform based on her experience using direct control myoelectric prostheses. The participant had congenital limb absence and could not reliably produce *tripod* grasp or index finger *point*, so she instead chose to produce a set of muscle contractions corresponding to wrist flexion, wrist extension, and rest (i.e., a relaxed muscle state). Within the classifiers, we mapped wrist flexion to *tripod*, wrist extension to *point*, and rest to *rest*.

#### 2.5.2 Socket Loading

The participant reported a deteriorated socket fit when all the distal components were attached. We therefore conducted classifier training using two separate loading conditions (Figure 1). Under the *load A* training condition, classifier training was performed with all the distal components attached to the socket (i.e., the TASKA Hand, wrist rotator, batteries, and on/off switch). Under the *load B* training condition**,** training was performed without the TASKA hand and wrist rotator attached to the socket. However, in this condition we temporarily attached a weight to the distal end of the socket equal to the weight of the participant’s clinically-prescribed prosthetic hand (Transcarpal Hand DMC Plus, Ottobock, Duderstadt, Germany). The attached weight was designed to make the resultant socket length approximately similar to the length of the participant’s clinically-prescribed prosthesis. We designed this loading condition to approximate the inertial loading and end-effector distance that the participant normally experiences using her prostheses.

#### 2.5.3 Arm Position

**FIGURE 2:**
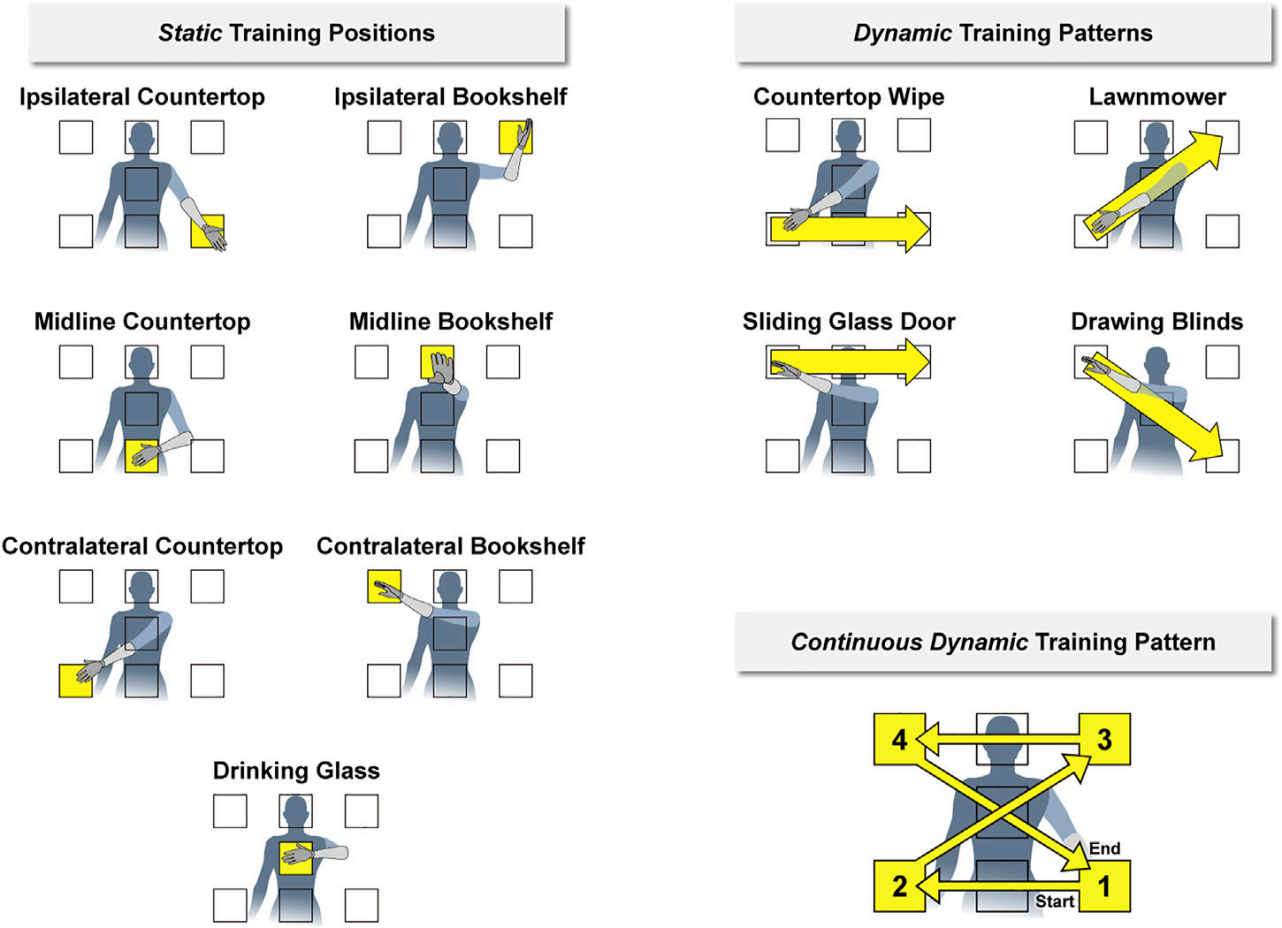
We imposed discrete arm positions and movement patterns during classifier training (shown here relative to the left arm). During the *static* condition, the participant held her hand for 5 seconds within seven different positions designed to cover a majority of the reachable workspace. During the *dynamic* condition, the participant moved her hand for 5 seconds between these positions in four different movement patterns. We also considered a *continuous dynamic* condition, in which the participant moved her hand throughout all positions during a single 20-sec movement pattern. The names of each position and pattern were based on a functional activity for that arm configuration. Corresponding positions and movement patterns were also established relative to the right arm (i.e., flipped horizontally).

We considered three classifier training strategies to account for changes to arm position during real-world prosthesis use (Figure 2). In the first strategy (i.e., the *static* training strategy), we recorded training data for the set of hand grasps while the participant held her arm still for 5 seconds in seven different positions. In the second strategy (i.e., the *dynamic* training strategy), we recorded training data for the set of hand grasps while the participant moved her arm for 5 seconds following four prescribed movement patterns. In the third strategy (i.e., the *continuous dynamic* training strategy), we recorded training data for a set of hand grasps while the participant moved her arm through all seven positions in a pre-defined 20-sec movement pattern. We chose this approach to minimize the total training time required and to reduce the potential impact of extraneous arm motion between data collection periods.

We used a naming convention for the seven arm positions and four movement patterns to make it easier for the participant quickly recognize and perform the requested action. The names were selected based on a functional task related to each position or pattern. The seven arm positions for the *static* training strategy were chosen to cover the majority of the reachable workspace:

1) **Ipsilateral Countertop**: as if reaching for an object located in front of and ipsilateral to the body at waist height
2) **Midline Countertop**: as if reaching for an object located directly in front of the body at waist height
3) **Contralateral Countertop**: as if reaching for an object located in front of and contralateral to the body at waist height
4) **Ipsilateral Bookshelf**: as if reaching for an object located in front of and ipsilateral to the body at head height
5) **Midline Bookshelf**: as if reaching for an object located directly in front of the body at head height
6) **Contralateral Bookshelf**: as if reaching for an object located in front of and contralateral to the body at head height
7) **Drinking Glass**: as if reaching for an object located directly in front of the body at sternum height

To maintain consistency between trials, the positions were defined relative to the participant’s anatomy and displayed on a wall in front of the participant at about an arm’s length away (Supplementary Figure S1).

The four prescribed movement patterns for the *dynamic* training strategy were chosen to cover the seven arm positions:

1) **Countertop Wipe**: moving from Contralateral Countertop to Midline Countertop to Ipsilateral Countertop in one fluid motion
2) **Lawnmower**: moving from Contralateral Countertop to Drinking Glass to Ipsilateral Bookshelf in one fluid motion
3) **Sliding Glass Door**: moving from Contralateral Bookshelf to Midline Bookshelf to Ipsilateral Bookshelf in one fluid motion
4) **Drawing Blinds**: moving from Contralateral Bookshelf to Drinking Glass to Ipsilateral Countertop in one fluid motion

The *continuous dynamic* training strategy was defined as a combination of the four movement patterns from the *dynamic* training strategy. However, the direction of the Countertop Wipe and Sliding Glass Door movement patterns were reversed so that the combined patterns could be performed continuously.

### 2.6 Offline Classification Performance

Before performing any real-time functional testing of the SMG-controlled prosthetic hand, we first examined the offline performance of classifiers using our different training strategies to account for socket loading and changes in arm positions.

#### 2.6.1 Training and Testing Data Collection

For a given set of training strategies, we collected classifier training and testing data during a single session. Two repeated sets of muscle contractions were collected for the set of hand grasps during each arm position or movement pattern. For example, under the *load A* training condition using the *static* training strategy, we collected two sets of ultrasound images over two 5-sec collection periods for muscle contractions corresponding to *tripod*, *point*, and *rest* for each of the seven *static* arm positions. Note that for our offline testing, we only considered the *static* and *dynamic* training strategies and did not consider the *continuous dynamic* training strategy.

The order of the selected hand grasps and arm positions or movement patterns were randomized. To avoid including any transient motion at the start of the recording, we instructed the participant to assume their initial arm position and then provided two audio cues (i.e., beeps). The first beep notified the participant that their prescribed arm motion would start in 3 seconds (i.e., a preparatory period). The second beep occurred 3 seconds later to notify the participant to initiate the prescribed arm motion (e.g., by holding their arm still for *static* training sessions or by moving their arm for *dynamic* training sessions). Ultrasound images were recorded at the end of the preparatory period and continued for 5 seconds, during which the participant maintained the requested muscle contraction. After the data collection session, data for two repeated sets of muscle contractions were randomly assigned as either classifier training data or classifier testing data.

#### 2.6.2 Offline Classification Accuracy Calculation

For a given set of training strategies, we built a series of LDA classifiers to predict a user’s desired hand grasp from the designated training data. For sessions using the *static* training strategy, we built seven classifiers using training data from each of the seven *static* positions individually and an eighth classifier from all seven *static* positions collectively. Similarly, for sessions using the *dynamic* training strategy, we built four classifiers using training data from each of the four *dynamic* movement patterns individually and a fifth classifier from all four *dynamic* patterns collectively.

We then calculated classification accuracy by inputting the designated testing data from each *static* position or *dynamic* movement pattern into the relevant classifier. This process generated a predicted grasp for each frame of testing data, which could be compared to the true grasp. Note that because the participant held only one grasp when recording a given dataset, the true grasp was the same for each frame in that dataset. Finally, classification accuracy was calculated by summing the number of correctly-predicted frames and dividing by the number of total frames in the dataset:

$$\mathrm{classification accuracy}\left(\%\right)=\frac{n_{\mathrm{correct predictions}}}{n_{\mathrm{total predictions}}}*100$$

Note that although data was collected for 5 seconds for both the *static* and *dynamic* training strategies, the total number of frames fluctuated between 57 or 58 across datasets.

#### 2.6.3 Characterizing Similarity of Ultrasound Images

We used the Structural Similarity Index (SSIM) to quantify differences in the ultrasound images acquired during classifier training. SSIM quantifies the similarity between two images by decomposing them into luminance, contrast, and structure components, which are compared separately between the two images. A final value between -1 and 1 is then computed as an index of similarity, where 1 represents perfect similarity. Given two images A and B , the index is defined as

$$\mathrm{SSIM}\left(A,B\right)={\left[l\left(A,B\right)\right]}^{\alpha }*{\left[c\left(A,B\right)\right]}^{\beta }*{\left[s\left(A,B\right)\right]}^{\gamma }$$

where l(A,B) , c (A,B) , and s(A,B) are the luminance, contrast, and structure components. The components are defined as

$$l\left(A,B\right)=\frac{2\mu_{A}\mu_{B}+C_{1}}{\mu_{A}^{2}+\mu_{B}^{2}+C_{1}}$$

$$c\left(A,B\right)=\frac{2\sigma_{A}\sigma_{B}+C_{2}}{\sigma_{A}^{2}+\sigma_{B}^{2}+C_{2}}$$

$$s\left(A,B\right)=\frac{\sigma_{\mathrm{AB}}+C_{3}}{\sigma_{A}\sigma_{B}+C_{3}}$$

where $\mu_{A}$ , $\mu_{B}$ , $\;\sigma_{A}$ , $\;\sigma_{B}$ , and $\sigma_{AB}$ are the local means, standard deviations, and cross-variance, and constant $C_{3}=C_{2}/2$ . The exponents are used to adjust the relevance of each component and were defined as α=β=γ=1 . To provide context to a calculated SSIM value, we determined the similarity of ultrasound images due to chance was 0.229. We determined the similarity due to chance as the average SSIM between 100 randomly generated ultrasound images (i.e., 4,950 unique comparisons). Each image was generated using random image intensity values from a Rayleigh distribution ( σ= 0.101 ) matching a representative ultrasound image of forearm muscle tissue.

To understand the consistency of ultrasound images for repeated conditions, we assessed the similarity of the ultrasound images for the two sets of contractions collected using the same classifier training strategies and hand grasp. Similarly, to understand the uniqueness of ultrasound images for differing hand grasps, we assessed the similarity of ultrasound images for two sets of contractions collected using the same classifier training strategies but different hand grasps. For all these comparisons, we calculated the SSIM within each frame of their 5-sec training periods. We then used a *t*-test to compare the average SSIM values between images using the same hand grasp and images using different hand grasps.

### 2.7 Real-Time Functional Performance

After offline classification testing, we conducted real-time functional testing using SMG to control the TASKA hand. Commands for the predicted hand grasp were delivered to the TASKA hand via Bluetooth. During functional testing, the MC Standard Wrist Rotator was fixed in place such that hand pronation or supination could not be controlled.

#### 2.7.1 Classifier Training

We collected a new set of training data to build classifiers for functional testing. We selected classifiers using different training strategies based on our evaluation of offline performance. By considering a smaller set of classifiers during functional testing we hoped to reduce the user’s burden of repeated functional testing under separate classifier strategies (e.g., to reduce fatigue). For the functional testing that involved classifiers trained using the *static* strategy, we included training data from all seven positions collectively. We also used a classifier built from training data collected using the *continuous dynamic* strategy. The functional tests were conducted immediately after training the relevant classifier (i.e., we did not train all classifiers before beginning the functional testing). Note that the TASKA hand was not attached when collecting training data during the *load B* training condition. However, the TASKA hand and related components were reattached to complete the functional testing.

#### 2.7.2 Prescribed Prosthetic Hand Grasps

**FIGURE 3:**
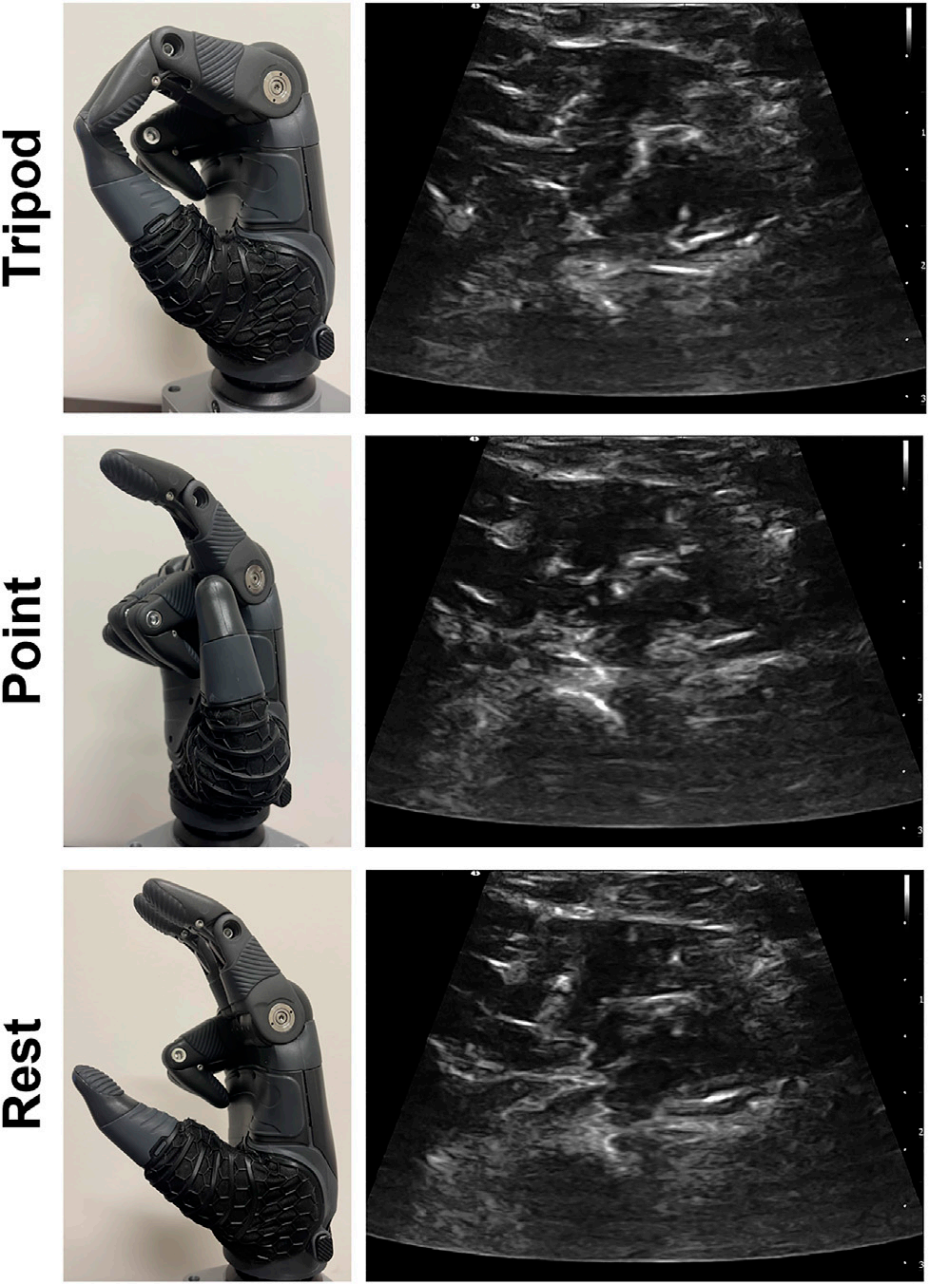
The TASKA hand was configured to include *tripod* grasp, index finger *point*, and *rest*. Each grasp was initiated in response to a different muscle activation pattern, including wrist flexion for *tripod*, wrist extension for *point*, and a relaxed muscle state for *rest*. Representative ultrasound images are included for each muscle activation pattern.

We configured the TASKA hand to perform the three grasps executed in classifier training, including *tripod*, *point*, and *rest* (Figure 3). We chose to implement these three grasps because only two of the grasps would be needed to perform the functional tests (*tripod* and *rest*) and one of the grasps would not be helpful (*point*). Each grasp was defined to be a discrete configuration of the thumb and fingers on the TASKA hand, such that a trained classifier initiated the TASKA hand to assume that configuration (i.e., the position and velocity of the thumb and fingers during grasp transitions were set in advance and not controlled by the classifier or participant). Thus, a participant could physically implement a grasp by activating their muscles in a manner that enabled the classifier to identify the desired grasp based on the ultrasound image of muscle deformation. We defined the *rest* grasp such that the thumb, index, and middle fingers of the TASKA hand were partially extended and the ring and little fingers were in a closed position (Figure 3). We used this definition to prevent the ring and little fingers from inadvertently moving blocks out of their designated rows during the targeted Box and Blocks Test, as described below. For consistency, we used this definition of *rest* grasp during all three functional tests.

#### 2.7.3 Functional Tests

We instructed the participant to perform three functional tests involving grasping and moving one-inch wooden blocks. Each functional test included a quantifiable score related to completion speed. We also recorded the participant’s performance with a video camera. Although the primary purpose was to ensure accurate quantification of the test scores, we also used comparisons of the recorded video for a general observational analysis of functional performance.

Additionally, we calculated two outcome measures to characterize the efficiency of grasp selection. First, we counted the “number of transient bouts” during each test. A transient bout was defined as an instance when the classifier predicted a grasp for less than five consecutive frames. Note that a transient bout does not necessarily indicate that a predicted grasp was misclassified, as it is possible for a user to select a grasp for a brief period (i.e., <5 frames) if desired. However, given the relatively slow, pick-and-place nature of each functional test, we consider it unlikely that a user would deliberately choose to switch grasps that quickly. This outcome measure thus serves as an indirect indicator of a classifier’s ability to predict a user’s intended grasp. Second, we quantified the percent of all frames that were classified as *point* during each functional test. Although *point* was not a helpful grasp during functional testing, we included it in the repertoire of available grasps so that we might examine a participant’s ability to select a desired grasp from a set of grasps. Because the participant should not be selecting *point* to accomplish the functional tasks, these instances of *point* also served as an indirect indicator of a classifier’s ability to predict a user’s intended grasp. Note that classification of *point* during a functional test does not necessarily indicate that a predicted grasp was misclassified, but we consider it unlikely for a user to voluntarily select this grasp when attempting to hold a block.

##### 2.7.3.1 Box and Blocks Test

**FIGURE 4:**
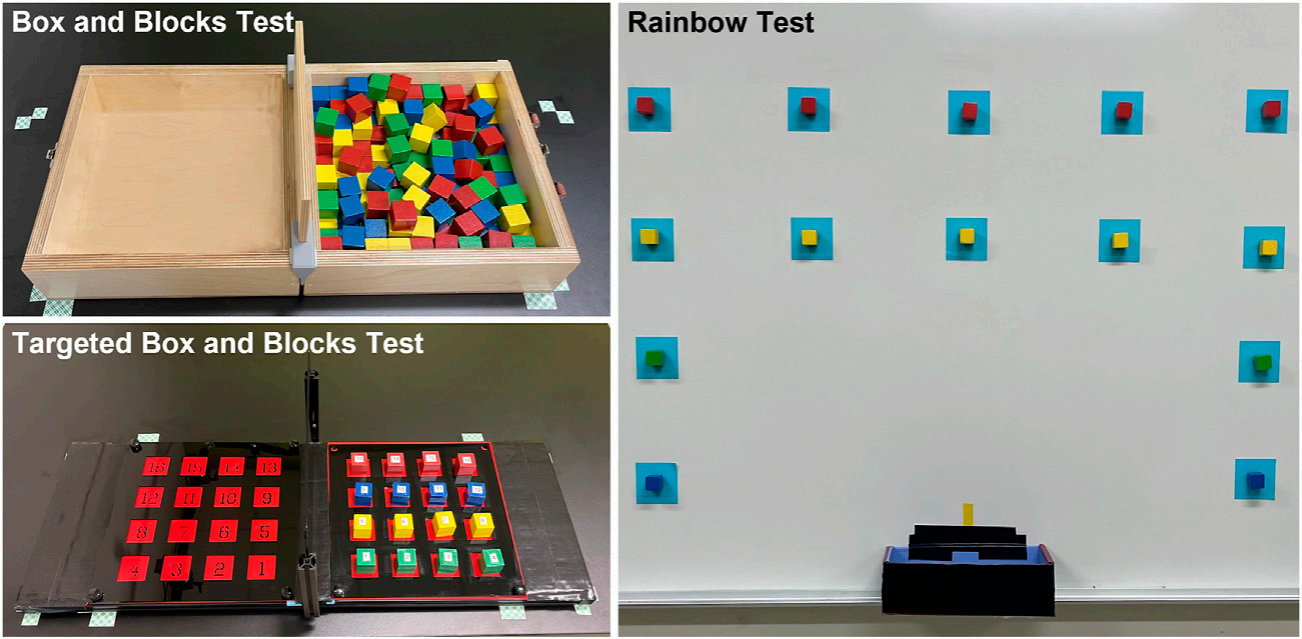
The three functional tasks included the Box and Blocks Test, the Targeted Box and Blocks Test, and the Rainbow Test.

The Box and Blocks Test (BBT) is a common measure of gross manual dexterity (Mathiowetz et al., 1985). The set-up consists of two 10-inch square compartments separated by a six-inch-tall partition (Figure 4). The compartment on the side of the arm being tested is filled with 150 one-inch wooden blocks, which are mixed such that the blocks rest in many different orientations. The test is scored by the number of blocks transported over the partition in 1 minute. Subjects may transport the blocks in any order as long as their fingertips cross the partition before releasing the block into the opposite compartment. The BBT apparatus was placed on a table set to 10 cm below the participant’s right anterior superior iliac spine. The apparatus was positioned 4 cm from the proximal edge of the table with the box partition aligned with the participant’s midline.

##### 2.7.3.2 Targeted Box and Blocks Test

The Targeted Box and Blocks Test (tBBT) is a modified version of the BBT involving only 16 blocks (Kontson et al., 2017). The blocks are placed in a four-by-four grid in the compartment on the side of the arm being tested (Figure 4). The blocks are numbered 1 to 16, beginning with the innermost block on the bottom row and moving across each row. Subjects must transport the blocks between the compartments in numbered order. Each block must be placed in its mirrored position in the other compartment. The test is scored by the time required to transport all 16 blocks. The tBBT was performed on the same table as the BBT with the apparatus placed in the same position described previously. Note that we made a minor modification to the tBBT apparatus by removing both compartments’ outer walls (Figure 4). Because of the large size of the TASKA Hand, it collided with the walls when the participant attempted to manipulate blocks located near the edge of the compartment. Since this study was intended to demonstrate the feasibility of using prostheses controlled by SMG during functional tasks, we felt it was appropriate to remove the walls so that the tBBT could be completed more easily.

##### 2.7.3.3 Rainbow Test

The Rainbow Test was developed for this study to evaluate grasp control over a wider variety of arm positions than is required by the BBT or tBBT (Figure 4, Supplementary Figure S2). A series of 14 squares were marked on a magnetic whiteboard following an approximate arch shape. One-inch magnetized blocks with magnets attached were placed inside each square. We instructed the participant to transport each block from the whiteboard to a collection box placed at waist height along the midline of the body. Blocks were transported in a designated order, beginning with the bottom blocks on the side ipsilateral to the prosthesis and continuing up each column. The test was scored by the time required to transport all blocks.

#### 2.7.4 Repeatability of Functional Performance

To assess whether the participant could repeatedly perform functional tasks using an SMG-controlled prosthetic hand, real-time functional performance was assessed for three separate scenarios:

1) *Short-term testing*: The participant trained a classifier and performed the set of three functional tests in random order. The participant then repeated the set of three functional tests (presented in a new random order) for a second and third time without retraining the classifier.
2) *Three-hour testing*: The participant trained a classifier and then wore the socket for 3 hours. Every 30 min, the participant performed the set of three functional tasks in random order. During each break between the functional testing, the TASKA hand was turned off and the participant was able to move freely and perform normal daily activities. Although the TASKA Hand could not be actively controlled during breaks, it could still be used for passive object manipulation. The participant performed pre-defined tasks during each break (Table 2), which were staggered to require increased arm movement and socket loading over time. For this testing scenario, we used a linear regression model to reveal any changes to the outcome measures over this 3-h period.
3) *Simulated donning/doffing*: The participant trained a classifier and performed the set of three functional tests in random order. Next, the ultrasound transducer was removed and replaced to simulate donning/doffing of the socket. The participant then repeated the set of three functional tests in random order without retraining the classifier.

**TABLE 2:** Activities performed during each break in the 3-h testing. All activities were performed with the arm wearing the sonomyographic prosthesis. The clinically-prescribed myoelectric prosthesis was worn on the contralateral arm for the bimanual activities. The TASKA hand was not turned on during these breaks, but the participant was able to passively manipulate objects using the hand.

Interval	Activities
0–30 min	• Typing on a keyboard
	• Short walks while allowing natural arm swing
30–60 min	• Typing on a keyboard
	• Short walks while allowing natural arm swing
60–90 min	• Typing on a keyboard
	• One-minute walk while allowing natural arm swing
	• Bending over as if to pick up a dropped object (1x)
90–120 min	• Typing on a keyboard
	• Three-minute walk while allowing natural arm swing, including going up and down one flight of stairs
120–150 min	• Typing on a keyboard
	• Bimanual lift of light (205 g) box from ground to shelf at head height (3x)
	• Bimanual lift of heavy (1.059 kg) box from shelf at waist height to shelf at head height (3x)
	• Writing on whiteboard with three columns of text aligned to the midline, ipsilateral shoulder, and contralateral shoulder
150–180 min	• Typing on a keyboard
	• Pushed door open (3x)
	• Pulled door closed (3x)
	• Pushed a 17.87 kg cart up and down hallway for a total of 60 feet, including turn one turn half (3x)
	• Picked up 1.86 kg bucket vertically from the ground (3x)
	• Carried 1.31 kg bag for 30 s with elbow flexed to 90°

### 2.8 System Latency

End-to end system latency was approximated as the delay between the onset of volitional finger flexion in an individual without limb loss and the corresponding onset of finger flexion on the TASKA hand. We manually identified the start of each movement based on the acceleration signals (Supplementary Figure S3), and these time differences were averaged over 5 cycles. The resulting value encompasses latency associated with data acquisition, data transfer, processing, and communication with the TASKA Hand via Bluetooth.

We also calculated the processing latency for the MATLAB-based classification algorithm using all datasets recorded during real-time functional testing (see Section 2.7). The difference in successive timestamps were averaged across all files to compute the processing latency. Lastly, we computed the classification throughput as the number of predictions divided by the total elapsed time, based on the real-time functional testing datasets (see Section 2.7).

## 3 Results

### 3.1 Offline Classification Performance

We collected offline classification testing data for the right arm only. We collected enough data to evaluate both the *static* and *dynamic* training strategies for the *load A* and the *load B* conditions.

#### 3.1.1 Classification Accuracy

**FIGURE 5:**
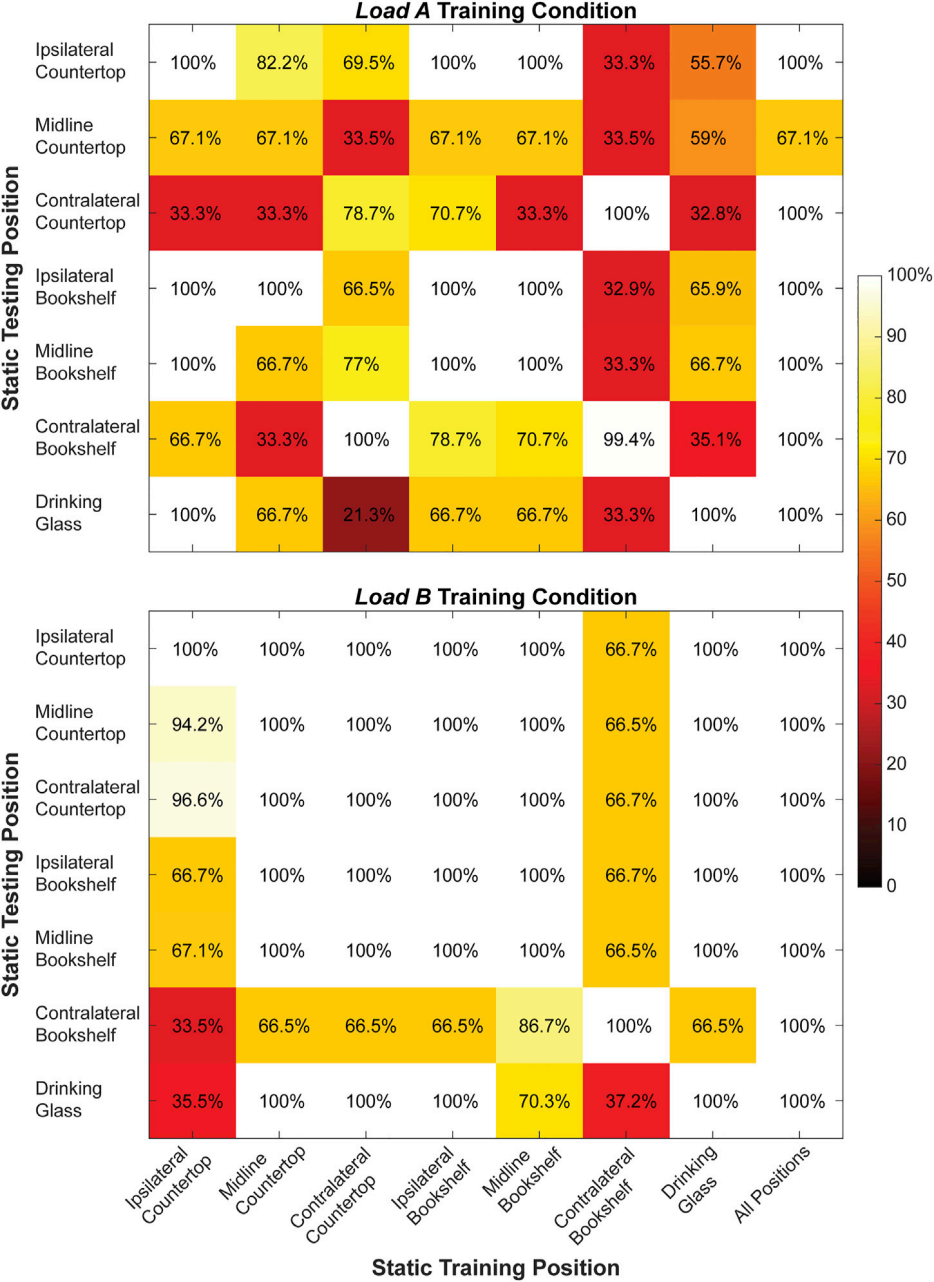
Classification accuracy (%) for the right arm when training and testing each of the static arm positions individually and from all static arm positions collectively during the *load A* and *load B* training conditions. Values shown in the main diagonal represent the intraposition classification accuracies, while the off-diagonal values represent the interposition classification accuracies.

**FIGURE 6:**
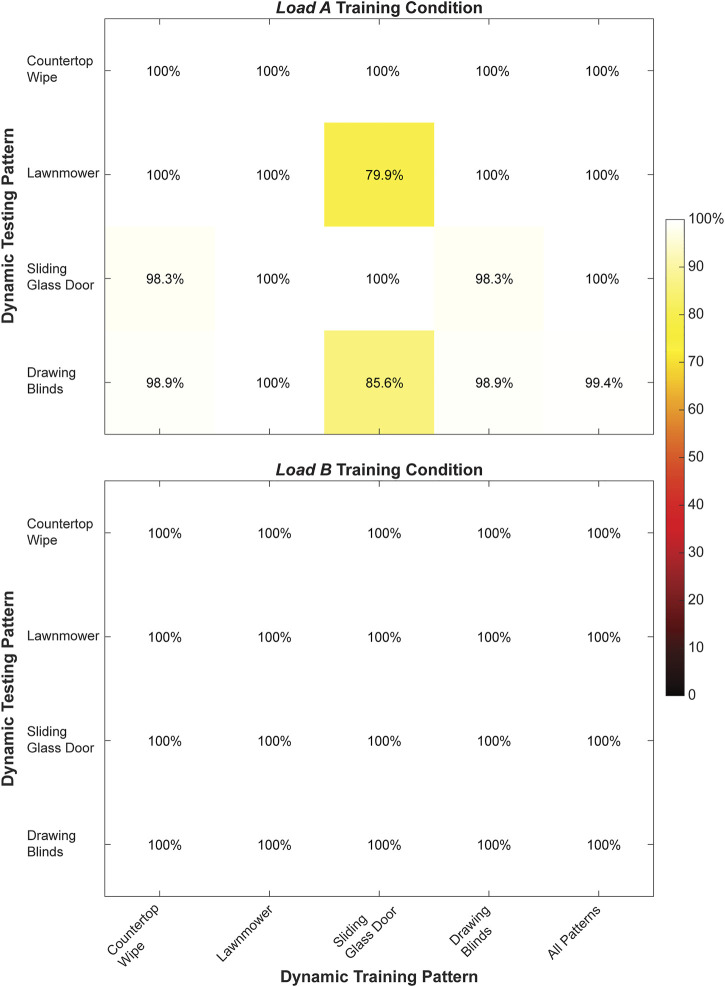
Classification accuracy (%) for the right arm when training and testing each of the *dynamic* movement patterns individually and from all *dynamic* movement patterns collectively during the *load A* and *load B* training conditions. Values shown in the main diagonal represent the intrapattern classification accuracies, while the off-diagonal values represent the interpattern classification accuracies.

We found that both the *static* training strategy (Figure 5) and the *dynamic* training strategy (Figure 6) could account for changes to arm position when predicting hand grasps during offline testing. However, offline classification accuracy was generally higher for the classifiers trained using the *dynamic* strategy than the *static* strategy. We also observed that offline classification accuracy was generally higher for classifiers trained under the *load B* condition compared to the *load A* condition.

During *static* testing, a classifier trained using all seven arm positions yielded the highest offline classification accuracy (*load A*: 95.3 ± 12.4%, *load B*: 100.0 ± 0.0%) compared to classifiers trained using a single arm position. Moreover, the average interposition classification accuracy (*load A*: 64.7 ± 25.6%, *load B*: 85.4 ± 20.3%) was lower compared to the average intraposition classification accuracy (*load A*: 92.2 ± 13.6%, *load B*: 100.0 ± 0.0%) for both loading conditions.

During *dynamic* testing, a classifier trained using all four movement patterns also had very high classification accuracy (*load A*: 99.9 ± 0.3%, *load B*: 100 ± 0.0%) compared to classifiers trained using single movement patterns. Notably, we observed 100% classification accuracy for every classifier trained using a single movement pattern under the *load B* training condition, as well as for the classifier trained using only the Lawnmower movement pattern under the *load A* training condition. Additionally, the average interposition classification accuracy (*load A*: 96.7 ± 6.7%, *load B*: 100.0 ± 0.0%) was lower compared to the average intraposition classification accuracy (*load A*: 99.7 ± 0.6%, *load B*: 100.0 ± 0.0%) for both loading conditions.

#### 3.1.2 Similarity of Ultrasound Images

**FIGURE 7:**
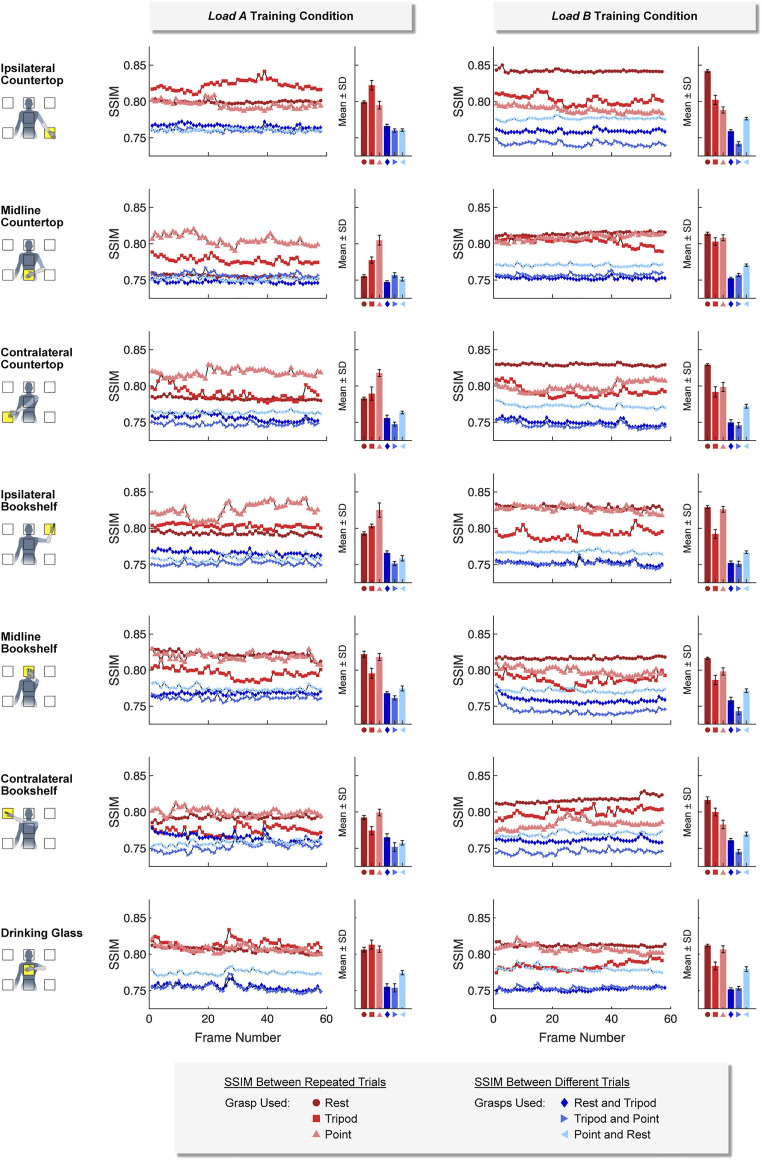
For the *static* training strategy, we found that ultrasound images collected using the same hand grasp (i.e., repeated trials) were more similar to each other than ultrasound images collected using different hand grasps (i.e., different trials). The Structural Similarity Index (SSIM) was calculated for each image frame of the respective 5-sec training period.

**FIGURE 8:**
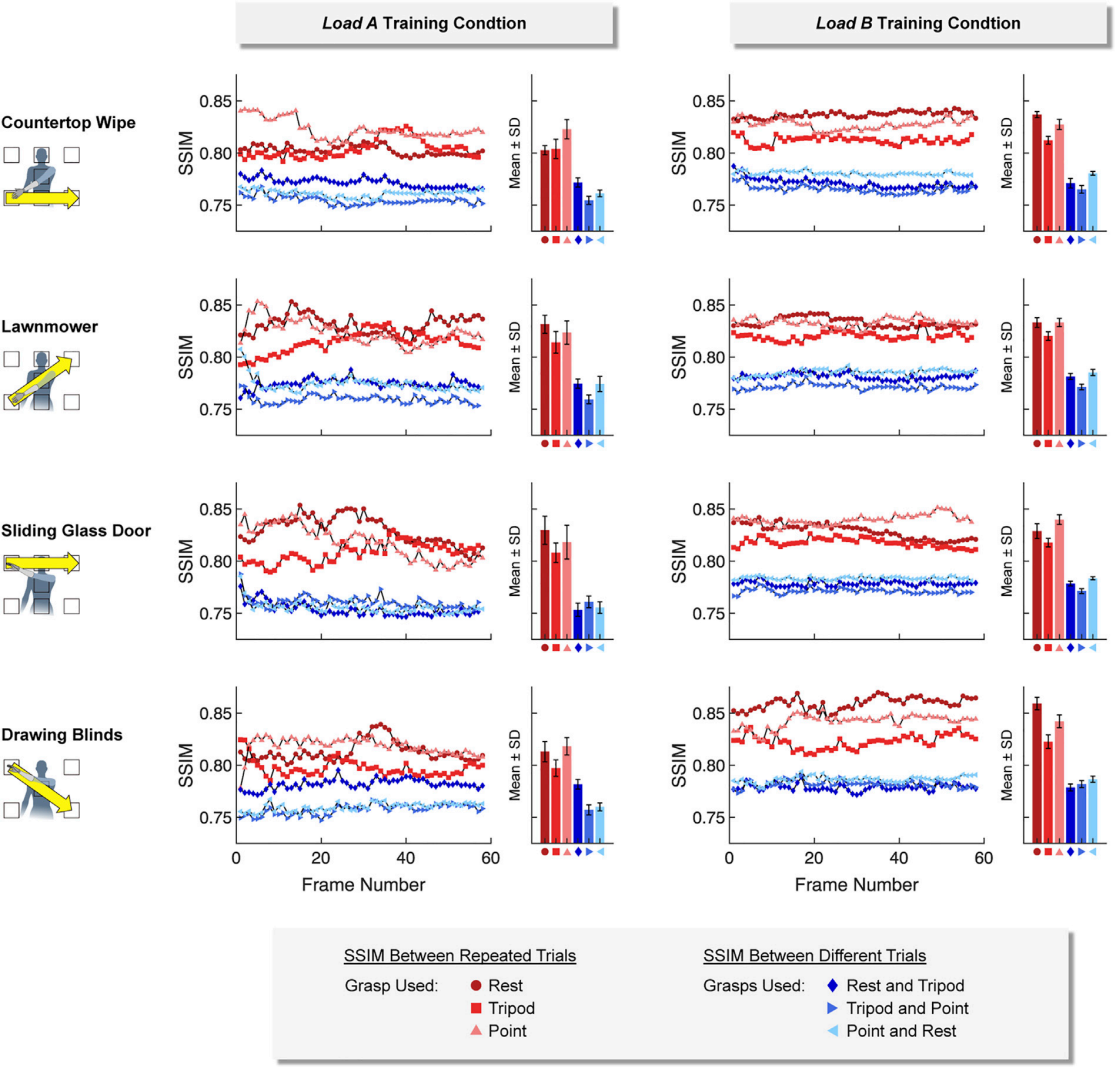
For the *dynamic* training strategy, we found that ultrasound images collected using the same hand grasp (i.e., repeated trials) were more similar to each other than ultrasound images collected using different hand grasps (i.e., different trials). The Structural Similarity Index (SSIM) was calculated for each image frame of the respective 5-sec training period.

Ultrasound images collected using the same hand grasps were more similar (i.e., a higher SSIM value) than ultrasound images collected using different hand grasps. This was true for images collected using both a *static* training strategy (Figure 7) and a *dynamic* training strategy (Figure 8). For example, images collected while using the same hand grasp during *static* training yielded an average SSIM of 0.800 ± 0.018 for *load A* and 0.806 ± 0.017 for *load B*. Images collected while using different hand grasps during *static* training yielded a significantly lower average SSIM of 0.759 ± 0.008 for *load A* (−5.1%, *p* < 0.001) and 0.759 ± 0.012 for *load B* (−5.9%, *p* < 0.001). Further, the images collected while using the same hand grasp during *dynamic* training yielded an average SSIM of 0.815 ± 0.015 for *load A* and 0.831 ± 0.013 for *load B*. Images collected while using different hand grasps during *dynamic* training yielded a significantly lower average SSIM of 0.764 ± 0.010 for *load A* (−6.3%, *p* < 0.001) and 0.778 ± 0.007 for *load B* (−6.4%, *p* < 0.001).

In addition, we observed that the difference between SSIM values using the same hand grasp and SSIM values using different hand grasps were more pronounced when using a *dynamic* training strategy compared to a *static* training strategy (i.e., the *dynamic* training could better differentiate images collected using the same hand grasp from images collected using different hand grasps). The average difference for SSIM values using a *dynamic* training strategy (*load A*: 0.0516 ± 0.0180; *load B*: 0.0532 ± 0.0162) were significantly higher than the average difference for SSIM values using a *static* training strategy (*load A*: 0.0403 ± 0.0183, *p* < 0.001; load B: 0.0475 ± 0.0223, *p* < 0.001).

We also observed that the SSIM values calculated for *load A* were more variable than the SSIM values calculated for *load B*. The average standard deviation for the SSIM values using the *dynamic* training strategy for *load A* (0.0074 ± 0.0034) was significantly higher than the average standard deviation for *load B* (0.0039 ± 0.0015, *p* < 0.001). However, the average standard deviation for SSIM values using the *static* training strategy were not significantly different between *load A* (0.0040 ± 0.0020) and *load B* (0.0036 ± 0.0017).

### 3.2 Real-Time Functional Performance

#### 3.2.1 Short-Term Testing

**FIGURE 9:**
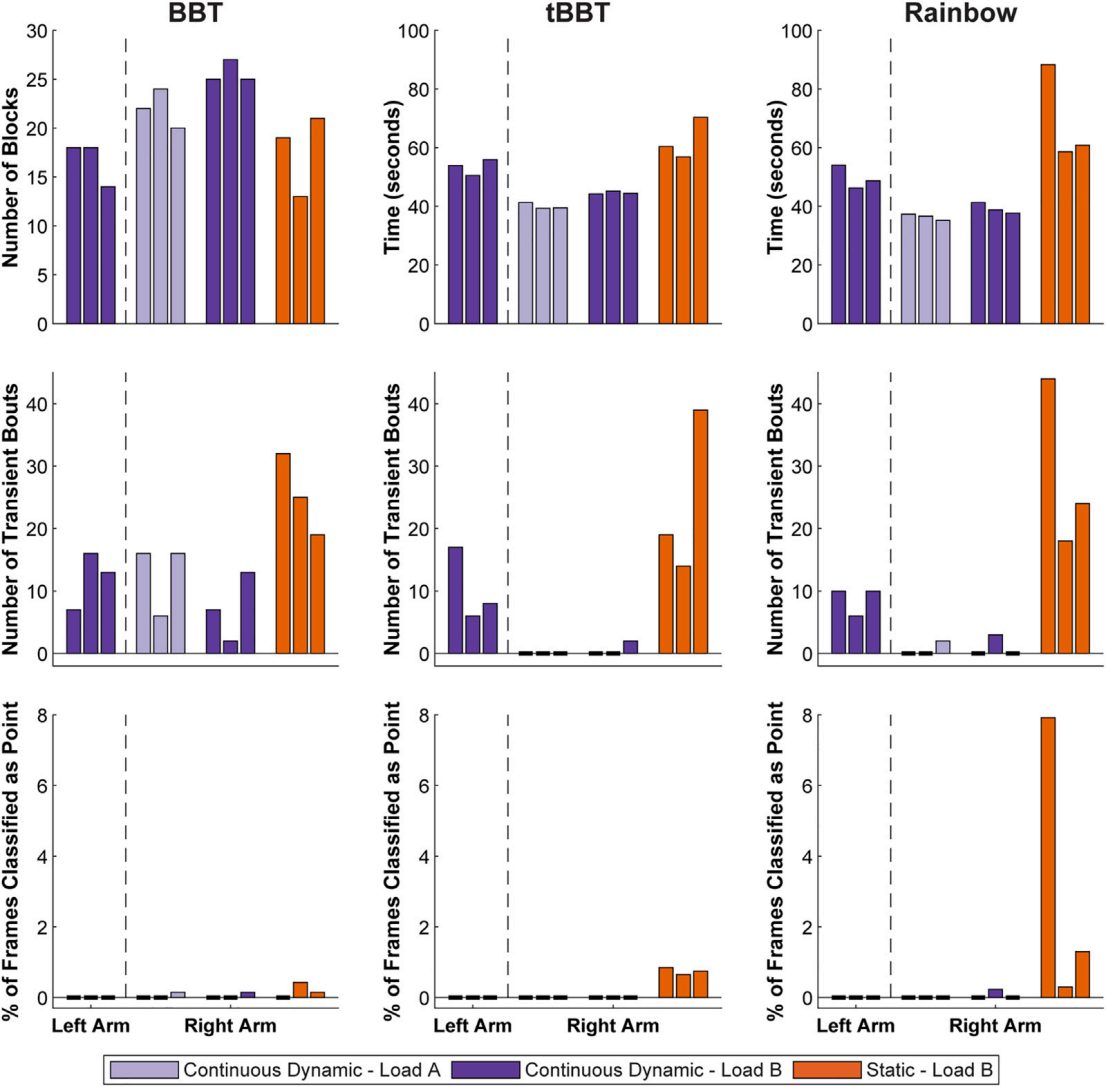
Functional outcome measures achieved during short-term testing. Testing with the left arm was completed using a classifier trained with a *continuous dynamic* strategy under the *load B* condition. Repeated testing with the right arm used *continuous dynamic* classifiers under the *load A* and *load B* conditions, as well as a *static* classifier under the *load B* condition (BBT = Box and Blocks Test; tBBT = Targeted Box and Blocks Test).

The participant successfully completed the short-term testing using both her left and right arms (Figure 9). Based on our evaluation of offline performance, we chose to conduct testing for the left arm using a classifier trained using only a *continuous dynamic* strategy under the *load B* condition. However, testing for the right arm included classifiers trained using a *continuous dynamic* strategy under both the *load A* and *load B* conditions, as well as a classifier trained using a *static* strategy under the *load A* condition.

Test scores for the left arm were relatively consistent across the three rounds of functional testing with the left arm (BBT: 16.7 ± 2.3 blocks, tBBT: 53.4 ± 2.7 s, Rainbow: 49.7 ± 4.0 s). No frames were ever classified as *point*, but there were some transient bouts (BBT: 12.0 ± 4.6 bouts, tBBT: 10.3 ± 5.9 bouts, Rainbow: 8.7 ± 2.3 bouts).

For the right arm, outcome measures were generally better when using a *continuous dynamic* training strategy than when using a *static* training strategy. For example, the participant moved more blocks on average during BBT for the *continuous dynamic* classifiers (*load A*: 22.0 ± 2.0 blocks, *load B*: 25.7 ± 1.2 blocks) than for the *static* classifier (*load B*: 17.7 ± 4.2 blocks). Completion times were also faster during tBBT for the *continuous dynamic* classifiers (*load A*: 40.0 ± 1.1 s, *load B*: 44.6 ± 0.5 s) than for the *static* classifier (*load B*: 62.5 ± 7.0 s). Similarly, the completions times were faster during the Rainbow test for the *continuous dynamic* classifiers (*load A*: 36.4 ± 1.1 s, *load B*: 39.3 ± 1.8 s) than the *static* classifier (*load B*: 69.3 ± 16.5 s). The mean number of transient bouts across all three rounds of functional testing were lower for the *continuous dynamic* classifiers (*load A*: 4.4 ± 6.8 bouts, *load B*: 3.0 ± 4.4 bouts) than for the *static* classifier (*load B*: 26.0 ± 10.2 bouts). Similarly, the mean percent of frames classified as *point* across all three rounds of functional testing were lower for the *continuous dynamic* classifiers (*load A*: 0.0 ± 0.1%, *load B*: 0.0 ± 0.1%) than for the *static* classifier (*load B*:1.4 ± 2.5%). However, these two metrics for the *continuous dynamic* classifier were generally similar between the *load A* and *load B* training conditions.

Observational analysis of functional testing yielded additional insights not captured by the quantifiable outcome measures. We observed that control of the prosthetic hand when using the *static* classifier was extremely sensitive to changes in arm position, as there were many instances where the hand closed to *tripod* grasp at improper times (Supplementary Videos S1–S3). When this occurred, the participant needed to slightly change her overall arm position to help the classifier identify a *rest* state and allow the hand to open. This behaviour created difficulties with both grasping and releasing blocks, especially during the Rainbow test when a wide range of arm positions were required. In contrast, the participant retained excellent control over the hand’s behaviour when using a *continuous dynamic* classifier, regardless of arm position (Supplementary Videos S4–S6).

#### 3.2.2 Three-Hour Testing

**FIGURE 10:**
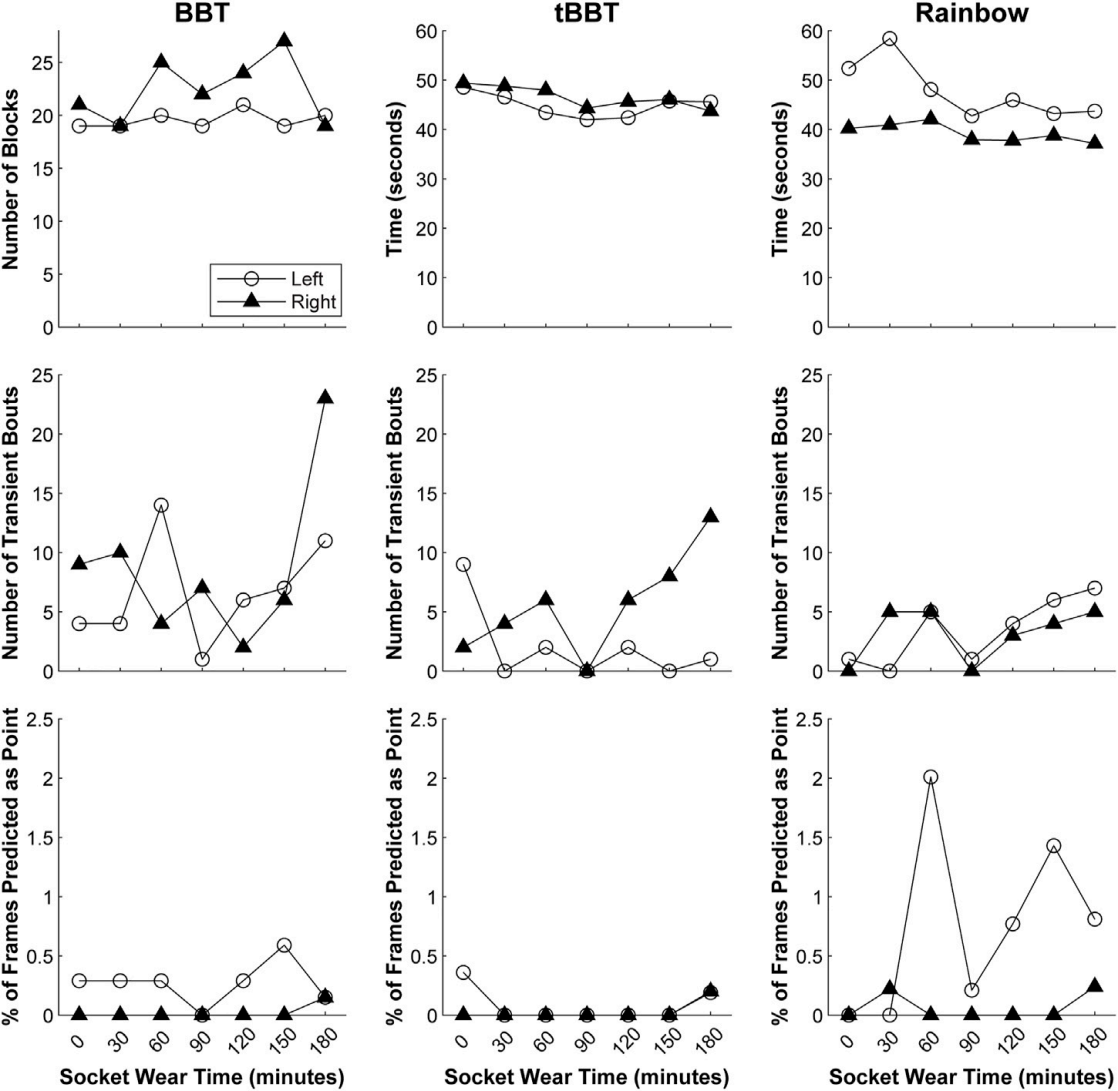
Functional outcome measures achieved during 3-h testing. Testing with both arms was completed using a classifier trained with a *continuous dynamic* strategy under the *load B* condition (BBT = Box and Blocks Test; tBBT = Targeted Box and Blocks Test).

The participant successfully completed the 3-h testing using both her left and right arms (Figure 10). Based on our evaluation of offline performance, we chose to only conduct testing with classifiers trained using a *continuous dynamic* strategy under the *load B* condition.

Our regression models revealed that although the outcome measures fluctuated slightly over time, they remained relatively stable as the participant actively moved her arm between testing intervals (Supplementary Table S1). However, the number of blocks moved during BBT with the left arm showed a small improvement over time (*p* = 0.038). Similarly, the completion time during tBBT with the right arm also slightly decreased over time (*p* = 0.011). We also observed there was a small increase in the number of transient bouts during the Rainbow test with the left arm (*p* = 0.027).

#### 3.2.3 Simulated Donning/Doffing

The participant successfully completed the simulated donning/doffing using both her left and right arms (Table 3). Similar to the 3-h testing, we chose to only conduct testing with classifiers trained using a *continuous dynamic* strategy under the *load B* condition.

**TABLE 3:** Functional outcome measures achieved during simulated donning/doffing. Testing with both arms was completed using a classifier trained with a *continuous dynamic* strategy under the *load B* condition (BBT = Box and Blocks Test; tBBT = Targeted Box and Blocks Test).

			Test Scores	Number of Transient Bouts	Percent of Frames Classified as *Point*
			Number of Blocks		
**BBT**	Left	Before removal	21	8	0
		After replacement	22	8	0
	Right	Before removal	23	1	1
		After replacement	26	0	0
			Time (seconds)		
**tBBT**	Left	Before removal	40.9	0	0
		After replacement	42.2	0	0
	Right	Before removal	43.9	0	0
		After replacement	43.6	0	0
**Rainbow**	Left	Before removal	37.4	2	0
		After replacement	36.8	0	0
	Right	Before removal	39.7	6	1.12
		After replacement	38.3	5	1.62

Changes in the outcome measures following transducer removal and replacement were minimal. The BBT scores for both arms increased by 1-3 blocks after the transducer was replaced, while the number of transient bouts and percent of frames classified as point decreased for the right arm only. The outcome measures did not consistently increase or decrease for tBBT and Rainbow. Completion times changed by 0.3–1.4 s, the number of transient bouts changed by 0–2 bouts, and the percent of frames classified as *point* changed by 0–0.5%.

### 3.3 System Latency

The average end-to-end latency (including data acquisition, data transfer, processing, and communication with the TASKA Hand via Bluetooth) was approximated as 532 ± 102 ms, while the latency for data transfer and the classification algorithm processing was 89.3 ± 8.4 ms. Classification throughput was measured to be 10.81 ± 0.12 predictions/sec.

## 4 Discussion

In this study, we report the first demonstration of an individual with upper limb loss using a prosthetic hand controlled by sonomyography (SMG) to perform functional tasks in real-time. We found that our participant could successfully complete three functional tasks that required selecting different hand grasps over a broad range of arm configurations. Additionally, the participant successfully repeated these tasks throughout 3 hours of use, as well as after removing and reattaching the ultrasound transducer. We also found evidence that training a classifier to predict hand grasps while moving the arm throughout the reachable workspace is a practical strategy for reducing misclassification related to changing arm position. This study supports the feasibility of using SMG to control upper limb prostheses in real-world applications, which ultimately may enable more intuitive control of multi-articulated prosthetic hands.

### 4.1 Real-Time Functional Performance

Real-time classification requires users to repeatably produce muscle contractions that are consistent with the signals used to train the classifier, and deviations in these contractions may cause the classifier to misidentify a user’s intended hand gesture. However, using a prosthesis in real-world settings involves complications that can degrade classification accuracy, such as muscle fatigue, muscle atrophy, fluctuating residual limb volume, perspiration (i.e., changes in electrode conductivity), changes in arm position, electrode shift, socket loading, and user adaptation or learning (Kyranou et al., 2018). Signal variability can consequently lead to classification failures and unpredictable behavior of a prosthetic hand, making it more challenging to use. Real-time functional testing with a physical prosthesis is therefore an essential step in the development and refinement of new control modalities. To this end, we are the first to show it is possible for individuals with upper limb loss to perform real-time functional tasks using an SMG-controlled prosthesis.

Our findings demonstrated that SMG could enable repeated completion of functional tests over a short-term testing period and over a 3-h testing period. For example, we demonstrated that a short 20-sec training sequence (per hand grasp) was sufficient to enable three consecutive hours of functional performance without retraining the classifier. Most functional outcomes were relatively stable throughout this period, although they improved with time in some cases (possibly via a learning effect). Interestingly, the number of transient bouts increased in one case, perhaps suggesting a decrease in classification accuracy that ultimately did not prevent completing the tasks.

We also found evidence that removal and replacement of the transducer between sequential tests minimally impacted real-time performance. Although more extensive testing is required, this finding may suggest that SMG classifiers may remain stable after doffing and redonning the socket. We chose to simulate donning and doffing for this feasibility study because performing these actions was uncomfortable and difficult for the participant to do. Additionally, the thermoplastic test sockets included a 3D printed bracket to hold the transducer and were not optimized to withstand repeated donning or doffing. However, sensor systems in clinical prosthesis sockets are permanently embedded. Thus, we believe effects of donning and doffing would be more appropriately explored in the future using a more robust socket design with permanently embedded transducers.

The three functional tests selected for this study required accurately selecting hand grasps over a broad range of arm positions. It should be noted that we developed the Rainbow test for this study, so it is not a previously established test of functional performance. We sought to examine functional performance over a user’s reachable workspace, but were unable to identify an established functional test with overhead and lateral reaching that we could easily incorporate. Because we did not include control of wrist rotation or flexion in our design, we were preventing from using established reaching tasks that require these actions, such as the Clothespin Relocation Test (Hussaini and Kyberd, 2017) or the Cubbies Task (Kuiken et al., 2016). Nonetheless, we were pleased to find our participant could complete the Rainbow test, as it involves manipulating small blocks with over a large range of arm movement. We encourage future studies to refine and implement the Rainbow test as an accessible measure of functional performance.

This study utilized a relatively simple hardware set-up to implement SMG control. As such, the participant was tethered to a tablet-based commercial ultrasound system that could not easily be transported. Further, we used a simple array transducer along with an LDA classifier that examined only single ultrasound images. We expect to see even better real-world performance when using a system optimized for SMG control that allows a user to move freely outside of a laboratory setting. In particular, we are encouraged by emerging technology involving single-element ultrasound transducers with miniaturized, low-power electronics that can be spatially distributed throughout a standalone prosthesis socket (Yang et al., 2019). We have previously shown that offline classification accuracy is not impacted by a sparse sensing approach (Akhlaghi et al., 2019), and anticipate this to be feasible for real-time testing in individuals with limb loss.

Another consequence of our simple hardware implementation was the considerable data processing latency between muscle contraction and resulting movement of the TASKA hand. The delay is largely attributable to our method for transferring the acquired ultrasound images to the classifier (i.e., using a USB-based video grabber to transfer images between the ultrasound system and the computer running MATLAB), combined with Bluetooth transmission from the computer to the TASKA hand. The participant reported noticing the latency and attempting to compensate for it during task performance, although we cannot quantify how successfully she was able to do this. For example, when she attempted to transition from *rest* to *tripod*, it was difficult for her to recognize whether the absence of immediate hand movement resulted from inherent system latency or actual misclassification. Further, we cannot quantify any small corrections she made to prompt the hand to move, such as changing arm positions or alternating between a relaxed and contracted muscle state (which might be detected as transient classification bouts). Thus, it is possible that our simple hardware implementation could have imposed a cognitive burden that slowed task completion times.

We acknowledge that the current system latency is nonoptimal. Based on our analysis, the time taken for data transfer and classification was only ∼16.7% of the total latency. Thus, the majority of the latency can be attributed to the latency for Bluetooth communication with the TASKA hand. We believe SMG-based prosthesis control will become more practical with continued hardware refinement and a hardwired communication with the TASKA hand. We are creating a more optimized implementation of SMG control using an integrated system, which we anticipate will have reduced data processing latency. The data acquisition latency can be reduced below 20 ms with a frame rate exceeding 50 frames/sec, as well as a communication and data transfer latency less than 10 ms. Thus, the overall latency will be under 125 ms, which has been reported as an optimal controller delay (Farrell and Weir, 2007).

### 4.2 Accounting for Changes in Arm Position

Importantly, our training strategies helped mitigate the effect of changing arm position. Arm position is a particularly concerning factor that may influence SMG signal variability because an individual’s arm position is biomechanically coupled to their forearm muscle activity. Users can recruit different combinations of muscles with varying force to counteract gravity and stabilize their arm in a particular orientation (Liu et al., 2014; Kyranou et al., 2018). Moreover, the shape and length of muscles in the residual limb can change depending on the joint angles of the entire arm (Fougner et al., 2011) and the compressive forces acting on the residual limb within the prosthesis socket (Hwang et al., 2017). Arm position not only impacts the relative position of muscles beneath skin-mounted sensors, but can also affect the contact force of sensors mounted within a socket (Stavdahl et al., 2020). This may be especially problematic for SMG control because any transducer shifting or change in contact force can drastically affect the imaging angle and thus the acquired ultrasound image. For these reasons, classification in an SMG-controlled prosthesis must be sufficiently robust under varying arm positions to achieve real-time functional performance.

Our strategies for training classifiers to account for changes to arm position were based on prior investigations of EMG-controlled upper limb protheses. For example, we implemented the established approach of recording training data from a variety of static arm positions (i.e., a *static* training strategy), which has consistently been shown to mitigate classification error for EMG-controlled systems when compared to using a single static arm position (Chen et al., 2011; Fougner et al., 2011; Scheme et al., 2011; Geng et al., 2012; Jiang et al., 2013; Khushaba et al., 2014, 2016; Liu et al., 2014; Hwang et al., 2017). However, we found this approach to be lengthy and fatiguing for the user, especially when a large number of arm positions are included (e.g., to cover the entirety of the user’s reachable workspace). Prosthesis users may tolerate such an approach in real-world settings if retraining is needed within or between days. Thus, we also chose to implement a second approach of generating training data during arm motion through a sequence of predefined positions (i.e., a *dynamic* training strategy). Prior studies of EMG control have found evidence that dynamic arm motions may reduce training time while also accounting for a variety of arm positions that might be involved during real-world prosthesis use (Scheme et al., 2011; Liu et al., 2012; Yang et al., 2017; Teh and Hargrove, 2020). In line with these past studies, we found that real-time performance during the short-term testing was better when using *dynamic* classifiers than with a *static* classifier trained using all seven *static* positions.

Our offline results also show that *dynamic* training strategies can have higher offline classification than *static* training strategies, suggesting this may be a preferred approach for a user to reliably select grasps in real-time settings. Although the *static* classifiers using a single arm position often had strong offline classification performance when tested with data from the same arm position used for training, the performance deteriorated when other arm positions were tested. Inclusion of all seven *static* positions in the training dataset improved the classification accuracy to nearly 100%. These findings are well-aligned with reports from the EMG literature showing a similar pattern of reduced inter-position classification accuracy compared to intra-position accuracy, as well as improved classification accuracy when training with multiple static arm positions. *Dynamic* classifiers were highly accurate when testing with different arm movements than those included in training. Inclusion of all four *dynamic* patterns yielded perfect classification accuracy. Again, similar results have been published previously for EMG control (Scheme et al., 2011; Liu et al., 2012; Yang et al., 2017; Teh and Hargrove, 2020). We also found that compared to the *static* training strategy, the *dynamic* training strategy could better differentiate ultrasound images collected using the same hand grasp from ultrasound images collected using different hand grasps.

Although we found that *dynamic* training strategies to be more effective than *static* training strategies, we did not attempt to determine the most optimal training sequence. The real-time performance was strong with a classifier trained using the 20-sec *continuous dynamic* strategy, but it is possible that shorter sequences would also work. Similarly, we only covered arm positions in the front and center of the body because most daily activities occur in this area. However, to achieve robust classification in real-world applications, classifier training might also consider regions lateral to the body, above the head, below the waist, or behind the body. Future work will be needed to explore what other training sequences are possible.

### 4.3 Accounting for Socket Loading

We observed that offline classification performance was partially dependent on socket loading during training. In general, offline classification accuracies were lower for the *load A* training conditions compared to the *load B* conditions. The increase in limb length, total weight, and distribution of weight introduced by the TASKA hand during *load A* training may have caused the socket to shift relative to the residual limb or induced muscle fatigue throughout the training process, leading to greater SMG signal variability. Although the socket was also loaded during *load B* training, the weight was smaller and located more proximally, which appeared to impact offline classification performance less significantly. Socket loading also impacted the ultrasound images of muscle deformation during the offline training sequences. In particular, there was increased variability in the similarity of ultrasound images during *load A* training compared to *load B* training. Again, this variability is likely due to the increased inertia from the mass of the TASKA hand located at an anatomically disproportionate distance from the elbow.

Since it is unlikely that a real-world prosthesis user would wear such a large hand relative to their body size, the poor offline classification performance and increased variability in muscle deformation patterns for the *load A* training should not be overly emphasized. We selected the TASKA hand prior to beginning this study because it could be easily integrated into our hardware implementation of SMG control, but we recognize it is not appropriately sized for many people. Unfortunately, the participant’s small stature and long residual limb meant that the hand was especially disproportionate. Inclusion of the *load B* training condition was meant to emulate a more realistic real-world setting in which the participant wore an appropriately sized hand and was not required to remove it prior to training (Note that although the TASKA hand can easily be removed and reconnected, this is not possible for all prosthetic hand models.) Nonetheless, it is very encouraging to note that real-time classification performance during the short-term testing with *load B* training was not substantially different from *load A* training. Even though the TASKA hand was not worn during *load B* training, the classifier seemed to tolerate any SMG signal variability from subsequent loading introduced by wearing the hand during testing. This finding underscores the importance of building classifiers using stable training data.

### 4.4 Limitations

Although this study demonstrates feasibility of the using SMG control a prosthetic hand in real-time, specific findings from this study should be interpreted cautiously given the inclusion of a single participant. We chose to include only one participant since the focus of this study was on the feasibility of using the technology, and not necessarily on the needs of a patient population. This choice allowed us to explore technical questions about the feasibility of our system, including multiple repetitions of experiments under different conditions, that would be challenging to do in a study involving many subjects. Including a single subject also facilitated our interpretation of the results, as the heterogeneous characteristics of individuals with upper limb loss can be a confounding factor in studies involving multiple participants. Nonetheless, optimizing the hardware, controller, and classifier to refine the implementation of SMG control for clinical use must be performed over a larger sample of users in future work.

It is also a limitation that our participant had congenital limb absence, as this restricted the number of distinct muscle contraction patterns she was able to produce (i.e., wrist flexion, wrist extension, rest). Our prior work has shown that many individuals with amputation can produce a higher number of distinct muscle contraction patterns corresponding to different hand gestures (rather than just wrist flexion and extension) and that these classes were successfully identified in offline testing using SMG (Dhawan et al., 2019; Engdahl et al., 2022). Future work should explore whether real-time classification performance remains accurate when an increased number of classified hand grasps are included.

Additionally, there may have been adaptation effects throughout the data collection process. The participant required some initial adjustment to unlearn the control strategy for her direct control myoelectric prostheses–namely, that the hand closed in response to wrist flexion, opened in response to wrist extension, and stopped moving when the muscles were relaxed. With the SMG prostheses, the hand moved to *tripod* in response to wrist flexion and opened when the muscles were relaxed. The participant anecdotally reported that she learned this control strategy quickly but occasionally forgot during testing. Adaptation issues may have persisted throughout testing because data collection occurred over several days, which meant that her sessions with SMG were interspersed with the use of her myoelectric prostheses. Thus, we cannot exclude the possibility that the outcomes gradually improved throughout a day of testing as she reacclimated to SMG control. We attempted to mitigate this by randomizing the order of the functional tests. However, because the participant was involved in the study development and piloting process, she had familiarity with the functional tests prior to formal data collection. It is possible that this prior practice helped her become more proficient at the tasks, so her performance may not be representative of more naïve users.

## 5 Conclusion

This study demonstrates the feasibility of using sonomyography (SMG) to control upper limb prostheses to complete functional tasks in real-time. Because ultrasound imaging enables spatiotemporal classification of both superficial and deep muscle activity, sonomyographic approaches may better account for the contributions of individual muscles than traditional myoelectric approaches for controlling prosthetic hands. Sonomyography is thus a promising modality for prosthetic control, and improved implementation of hardware and controller designs might enable increased functional performance and more intuitive control of prosthetic hands.

## Data Availability

The raw data supporting the conclusions of this article will be made available by the authors, without undue reservation.
