# Supplementary material for: First Demonstration of Functional Task Performance Using a Sonomyographic Prosthesis: A Case Study
Source: Front Bioeng Biotechnol. 2022 May 4;10:876836. doi: 10.3389/fbioe.2022.876836 (PMC9114778; doi:10.3389/fbioe.2022.876836)

Supplementary Material

Supplementary Table 1. Effects of socket wear time on the outcome metrics during the *three-hour testing*. Bold text indicates statistical significance.

	Outcome Metric	Slope	R^2	t-value	p-value
		LEFT ARM			
**BBT**	Number of Blocks	4.80×10^{-3}	0.154	0.954	**0.038**
	Number of Transient Bouts	2.26×10^{-2}	0.108	0.778	0.472
	Percent of Frames Classified as *Point*	2.00×10^{-4}	0.001	0.177	0.868
**tBBT**	Completion Time (seconds)	-1.37×10^{-2}	0.139	-0.897	0.411
	Number of Transient Bouts	-2.86×10^{-2}	0.332	-1.576	0.176
	Percent of Frames Classified as *Point*	6.07×10^{-4}	0.076	-0.641	0.550
**Rainbow**	Completion Time (seconds)	-6.96×10^{-2}	0.610	-2.797	0.381
	Number of Transient Bouts	3.45×10^{-2}	0.657	3.095	**0.027**
	Percent of Frames Classified as *Point*	4.82×10^{-3}	0.169	1.008	0.360
		RIGHT ARM			
**BBT**	Number of Blocks	1.070×10^{-2}	0.052	0.523	0.632
	Number of Transient Bouts	3.810×10^{-2}	0.129	0.861	0.429
	Percent of Frames Classified as *Point*	5.360×10^{-4}	0.375	1.732	0.144
**tBBT**	Completion Time (seconds)	-2.950×10^{-2}	0.753	-3.902	**0.011**
	Number of Transient Bouts	4.880×10^{-2}	0.557	2.509	0.054
	Percent of Frames Classified as *Point*	7.140×10^{-4}	0.375	1.732	0.144
**Rainbow**	Completion Time (seconds)	-2.120×10^{-2}	0.559	-2.519	0.053
	Number of Transient Bouts	1.310×10^{-2}	0.140	0.902	0.408
	Percent of Frames Classified as *Point*	3.330×10^{-4}	0.037	0.438	0.680

Supplementary Figure 1. Locations of the seven positions used during *static* training. Dimensions were measured from the center of each 10 x 10 cm square. The rows were located at the participant's head height, sternum height, and anterior superior iliac spine (ASIS) height. The columns were separated by a distance equivalent to the participant's shoulder width (30.5 cm).

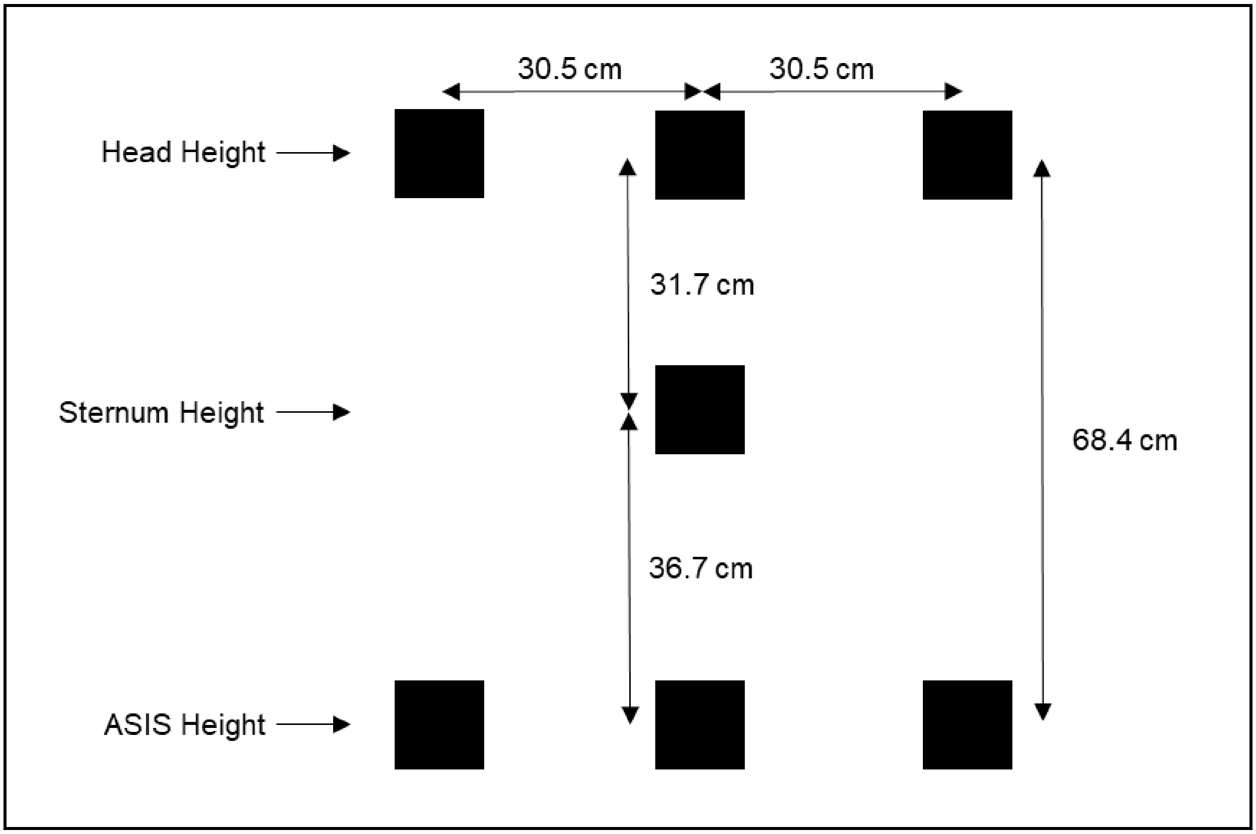

Supplementary Figure 2. Dimensions for the Rainbow Test were measured from the center of each 7.62 x 7.62 cm square. The collection box was placed at the height of the participant's anterior superior iliac spine.

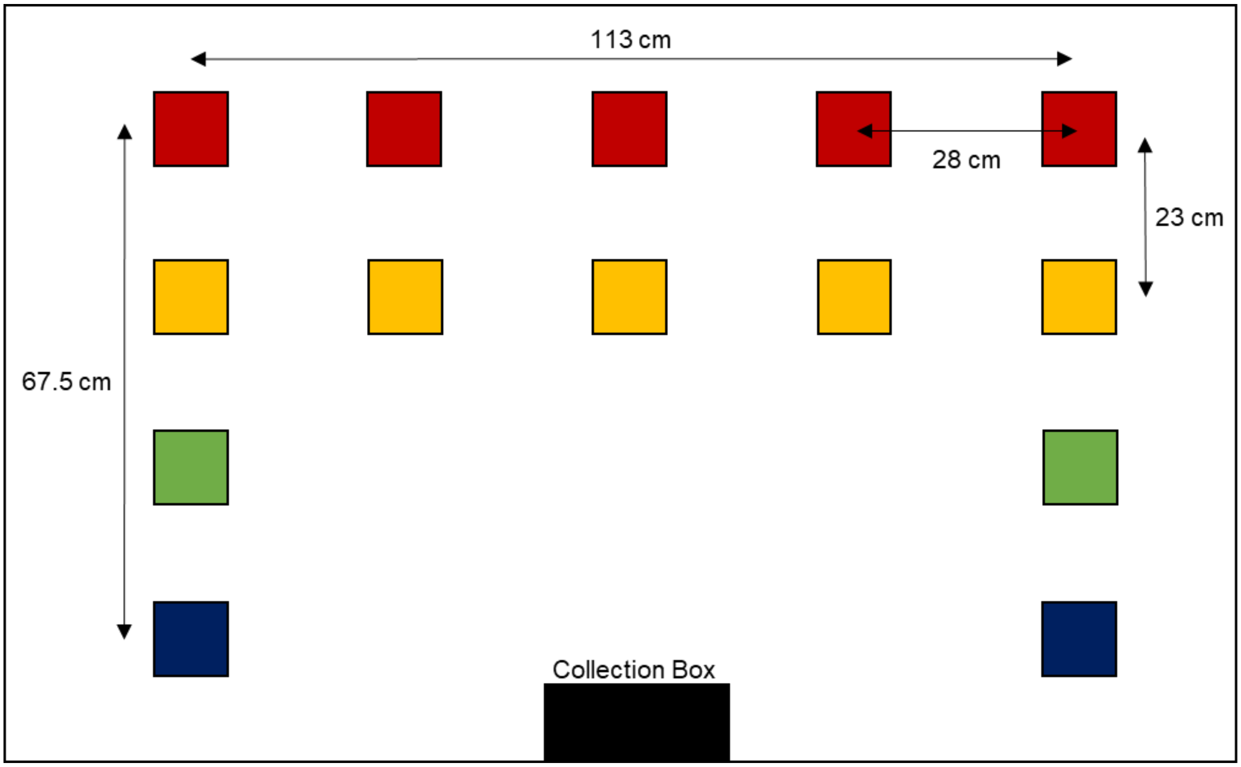

Supplementary Figure 3. Total latency from volitional muscle activation to TASKA prosthesis activation was approximated using two accelerometers. One accelerometer was placed on an able-bodied individual's index finger and the other was placed on TASKA hand's index finger in similar orientations. A classifier was trained on index finger flexion and the time difference between movement onsets was obtained from the z-axis acceleration based on visual inspection. A sample latency point extracted from the data is shown in plot.

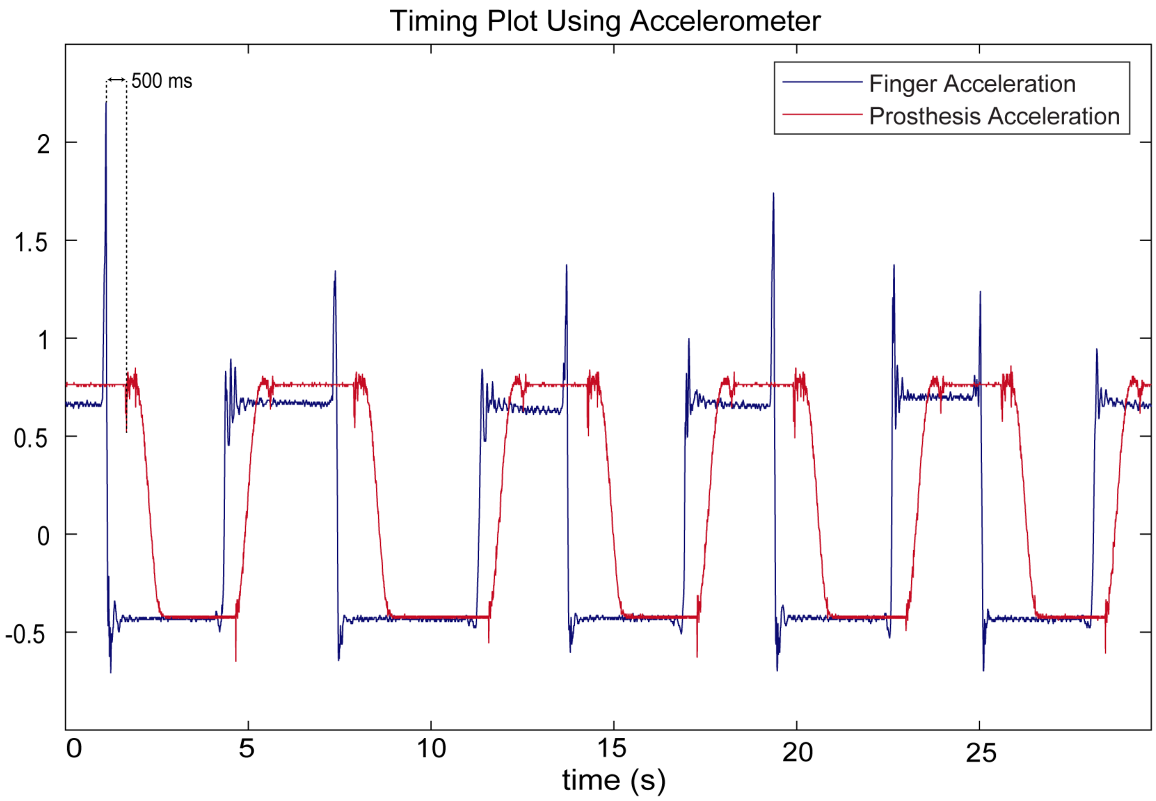

Supplement: Supplementary file 2 [file DataSheet1.PDF]
